# Epigenetic modification brings new opportunities for gene capture by transposable elements in allopolyploid *Brassica napus*

**DOI:** 10.1093/hr/uhaf028

**Published:** 2025-01-27

**Authors:** Yafang Xiao, Mengdi Li, Jianbo Wang

**Affiliations:** State Key Laboratory of Hybrid Rice, College of Life Sciences, Wuhan University, Wuhan 430072, China; State Key Laboratory of Hybrid Rice, College of Life Sciences, Wuhan University, Wuhan 430072, China; State Key Laboratory of Hybrid Rice, College of Life Sciences, Wuhan University, Wuhan 430072, China

## Abstract

Polyploids are widespread in plants and are important drivers for evolution and biodiversity. Allopolyploidy activates transposable elements (TEs) and causes genomic shock. Plant genomes can regulate gene expression by changing the epigenetic modification of TEs, but the mechanism for TEs to capture genes remains to be explored. *Helitron* TEs used the ‘peel-and-paste’ mechanism to achieve gene capture. We identified 3156 capture events and 326 donor genes of *Helitron* TEs in *Brassica napus* (A_n_A_n_C_n_C_n_). The donor genes captured by TEs were related to the number, length, and location of their exons. The gene-capturing TEs carrying donor gene fragments were evenly distributed on the genome, and more than half of them were involved in the construction of pseudogenes, becoming the reserve force for polyploid evolution. Gene fragment copies enhanced information storage, providing opportunities for gene mutation and the formation of new genes. Simultaneously, the siRNAs targeting TEs may act on the donor genes due to siRNA crosstalk, and the gene methylation levels increased and the expression levels decreased. The genome sought a balance between sacrificing donor gene expression and silencing TEs, allowing TEs to hide in the genome. In addition, epigenetic modifications may temporarily relax the control of gene capture during allopolyploidization. Our study identified and characterized gene capture events in *B. napus*, analyzed the effects of DNA methylation and siRNA on gene capture events, and explored the regulation mechanism of gene expression by TE epigenetic modification during allopolyploidization, which will contribute to understanding the formation and evolution mechanism of allopolyploidy.

## Introduction

Polyploidy is widespread in eukaryotes, and it affects genes, cells, and even entire ecosystems [1]. As an important driver of evolution, polyploidy may bring stronger environmental stress adaptability and ecological niche expansion to plants [2]. Due to the impact of hybridization and polyploidization, transposable elements (TEs) in the allopolyploid genome are activated [3], and then the difference of their content and density influence the establishment of the dominant subgenome [4]. Dealing with gene expression disorder and meiosis abnormality is the primary task for new polyploids after genome remodeling [5].

TEs are important contributors to the composition of plant genomes, accounting for 83.95% of the maize genome, 53.9% of the rice genome, and 53.78% of the *Brassica rapa* genome [6–8]. The variation of TE content among closely related species is drastic, and the difference is considered to be an important cause of genomic variation [7, 9]. TEs are also an essential part of the centromere sequences, and the amplification of LTRs in the *B. rapa* genome drives the evolution of centromeres [8]. Plants can achieve gene remodeling, induce phenotypic variation, and form new traits through TE insertion [10]. Retrotransposon insertions upstream of the *MdMYB1* gene in apple are involved in the red coloration of fruit skin as enhancers of gene expression [9]. Specific insertions of DNA transposon *MITEs* into *Arabidopsis B_sister_* (*ABS*) genes in *Arabidopsis thaliana* enable the achievement of precise regulation of gene expression [11]. Active TE insertions affect gene expression and threaten genome stability. To maintain genome stability, plant genomes devoted so much effort to silence TEs. DNA methylation is a widely adopted epigenetic modification for TE silencing in plants, and the main mechanism is the RNA-directed DNA methylation (RdDM) pathway. The RdDM pathway begins from the recruitment of RNA polymerase IV (Pol IV), which relies on 24 nt-siRNA to mediate the establishment and maintenance of DNA methylation [12]. The 24 nt-siRNA is enriched in germ cells, and CHH methylation accumulates during embryo development, which multiple methylation pathways target TEs to silence potentially harmful TE expression [13, 14]. Epigenetic modifications of silent TEs are present in generations and may occur in epigenetic reprogramming during sexual reproduction [15]. The genome can also regulate the neighboring gene expression by changing the siRNA abundance and DNA methylation levels of TE, and control stress resistance and tillering of rice [16, 17]. The sRNA transcribed by TEs can promote silencing, and the differences in the content and methylation levels of TEs may lead to the asymmetry between subgenomes in *Brassica napus* [18].

Despite the strong suppression and defense through epigenetic modifications that have been adopted, TEs still escape silencing and can proliferate. In contrast to the extensive research on the function and epigenetic modification of TEs near genes, the relationship between genes and TEs over long distances remains to be explored. Most TEs have the ability to capture gene fragments, and the phenomenon of gene capture has been found in animals and plants [19, 20]. Protein-coding genes (1560) were found to be captured by 2967 *Pack-MULE* TEs in rice [20]. Unlike ordinary DNA transposons, *Helitron* employs replicative rolling-circle mechanism that leaves the original template unaltered. When the 3′ termination signal is accidental malfunction or deletion, *Helitron* is able to capture the genome fragment and carry the captured sequence to a new site for recapture [21]. *Helitron* TE plays an important role in capture events of genome due to its unique structure and replication mechanism, leading to an increase in gene sequence copies and promoting genome evolution. The capture and insertion of TEs lead to the redistribution of chromosome sequences, gene fragment duplication, and dramatic change of local GC base content [20]. Meanwhile, capture events lead to the possibility that siRNA targeting TEs in the RdDM pathway act on their homologous gene fragments, resulting in epigenetic modification conflicts in the genome [22].

The *Brassica* genus is economically important and contains a large number of vegetable and oilseed crops. The U triangle including three diploid and three allopolyploid species in *Brassica* is a classic model for polyploid evolution studies [23]. Approximately 7500 years ago, *B. rapa* (A_r_A_r_) and *Brassica oleracea* (C_o_C_o_) underwent hybridization and polyploidization, and the two genomes gave rise to the new allopolyploid *B. napus* (A_n_A_n_C_n_C_n_) [24]. The functions of TEs in genome expansion and genomic asymmetry in *B. napus* have been widely discussed in recent years [18, 25]. However, the gene capture mechanism of TEs in allopolyploidization remains to be explored. *Helitron* has absolute quantity advantage and capture strength in *B. napus* compared with other plants. In our previous study, we found that the *Helitron* superfamily had the highest number of TEs in *B. napus* [26]. We revealed that TEs located within 2 kb upstream and downstream of genes can regulate gene expression levels through changes of epigenetic modifications, and influence the establishment of subgenome dominance by synergistic regulation of genomic asymmetric histone and siRNA mediated DNA methylation. Here, we further analyzed the characteristics and evolution fate of gene capture by *Helitron* TEs in *B. napus* genome, and combined siRNA and DNA methylation to construct the epigenetic modification maps of capture events in natural *B. napus*, resynthesized *B. napus*, and *in silico* ‘hybrid’ between *B. rapa* and *B. oleracea*. This study reveals the important role played by epigenetic modification of TEs in gene capture events during the process of allopolyploidization.

## Results

### Identification of gene-capturing *Helitron* TEs and donor genes

The ‘peel-and-paste’ replication mechanism of *Helitron* TEs determined that they had the strong ability to capture genes [21]. In our previous study, a total of 350 641 *Helitrons* were identified, which was the superfamily with the largest number of TEs in *B. napus* genome [26]. We further analyzed the *Helitrons* capturing gene fragment events at the whole-genome level in *B. napus* based on the *Helitrons* identified. To explore the influence of TEs and their epigenetic modification on gene capture, we excluded them with physical overlaps on chromosomes, and then performed BLASTN comparisons of the sequences of the remaining TEs and gene exons. Using the strict *E-value* (1 × 10^−40^) as the standard, a total of 3156 capture events and 1793 gene-capturing TEs were identified. According to the number of capture events occurring on TEs, they can be divided into single and multiple gene-capturing TEs. More than half (64.75%) of the TEs captured only one exon of some genes, while 35.25% of gene-capturing TEs conducted multiple capture events and carried two or more fragments. Meanwhile, the average length of multiple gene-capturing TEs was significantly longer than that of single (Fig. S1A). TEs carrying the captured gene fragments may recapture new genes, and the captured sequence length was continuously accumulated. When more than one gene sequence had high similarity to the same TEs, the gene with the highest bit score was considered to be the true donor, and different genes with the same *E-value* and bit score were all retained. Most of the genes in *B. napus* genome were free genes (without capturing gene fragments), and only a small part was captured by TEs. We identified a total of 326 donor genes from 539 captured genes and 100 501 free genes in *B. napus* genome, and the length of the captured sequences was a minimum of 92 bp, a maximum of 1270 bp, and an average of 230 bp.

### Characterization of donor genes and gene-capturing TEs

**Figure 1:**
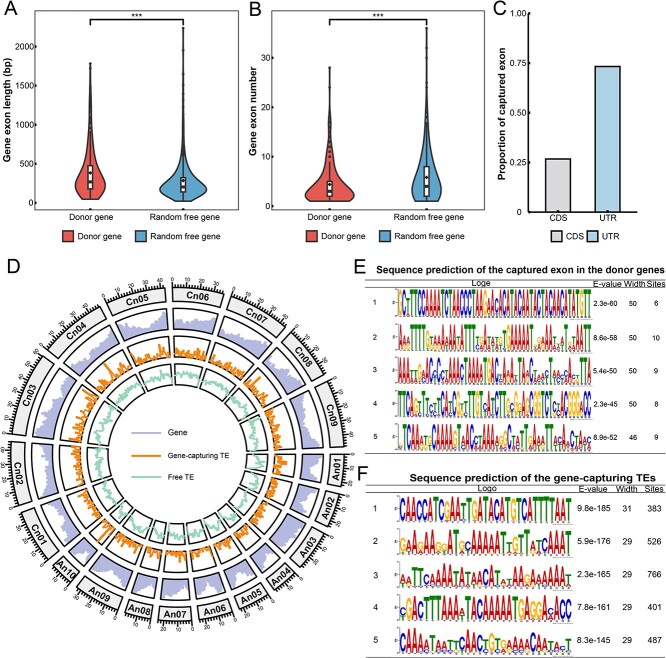
Characteristics of donor genes and gene-capturing TEs in *B. napus*. (A) Length of donor and random free gene exons. (B) The number of donor and random free gene exons. The squares represented the mean value. Significant differences between donor and free gene exons, using the Wilcoxon test (^***^P < 0.001). (C) The proportion of captured exons. (D) Distribution density of all genes, gene-capturing TEs and free TEs on chromosomes of *B. napus* genome. (E) Repeat motif prediction of captured exons. (F) Repeat motif prediction of gene-capturing TEs.

To explore the preference for capture event occurrence, we further analyzed the characterization of donor genes and gene-capturing TEs. The length of donor gene was similar to that of free gene, and there was no significant difference (Fig. S1B). For accurate and clear display, 326 (the same number as the donor genes) free genes were randomly selected for exon length and number statistics. Donor gene exons were significantly longer than random free genes (Fig. 1A), while the exon number had a significant advantage in the random free genes (Fig. 1B). Longer gene sequences may be able to provide more opportunities for TEs to replicate. Meanwhile, we also analyzed the types of captured sequences in the capture events and found that most of the captured exons (73.26%) were untranslated regions (UTRs, Fig. 1C), implying that protein-coding regions may not be the direct purpose of capture. In contrast to the UTRs, which had a clear tendency to be captured, the density distribution of capture and free (without carrying gene fragments) TEs was similar, and mostly evenly distributed on chromosomes (Fig. 1D). The average length of the gene-capturing TEs was significantly longer than that of the free TEs, and the GC content of their sequences was similar (Table S1). In order to further explore the sequence characteristics prone to capture events, we performed motif enrichment for sequences of the donor gene captured exons and gene-capturing TEs, and we found that the captured regions contained conserved motifs that all had a large number of continuously repeating arrangement A/T bases (Fig. 1E and F). The *Helitron* TEs of *B. napus* had an obvious tendency to integrate gene fragments in the AT nucleotide region in the genome.

### TEs carrying captured gene fragments evenly distributed in the genome

**Figure 2:**
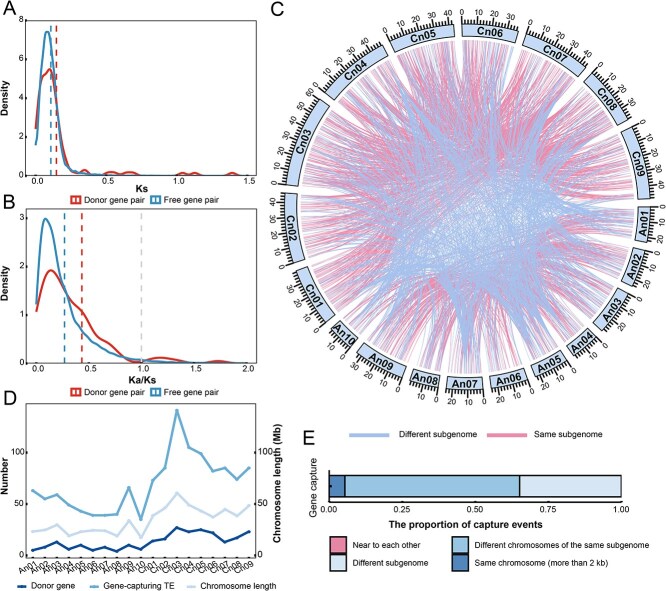
The fate of genes captured by TEs. (A) The synonymous substitution values of homoeologous genes. (B) The Ka/Ks ratios of the homoeologous gene pairs. (C) The position relationship between gene exons and TEs during capture events. (D) The relationship between the number of donor genes and gene-capturing TEs, and the chromosome length. The Y-axis on the left side of the graph represented the number of donor genes and gene-capturing TEs, and the chromosome length was displayed on the right. (E) The proportion of the relationship between the gene-capturing TEs and captured regions.

To understand the effect of selection pressure on capture events, we divided the 29 976 homoeologous gene pairs [27] of *B. napus* genome into donor and free gene pairs, and calculated the nonsynonymous (*Ka*) and synonymous (*Ks*) values and *Ka*/*Ks* ratios. Synonymous substitution did not affect the amino acid sequence, so *Ks* was often regarded as a molecular clock. The *Ks* values of donor gene pairs were higher than that of the free gene pairs, which may imply that the genes with longer mutation times were selected as donor genes (Fig. 2A). Furthermore, the *Ka*/*Ks* ratios of both donor and free homoeologous gene pairs were <1, which indicated they were undergoing purifying selection (Fig. 2B). However, the *Ka*/*Ks* ratios of donor gene pairs were higher than those of free gene pairs, and the donor gene preferred to be served by genes with more relaxed selection (Fig. 2B).

With a focus on the destination and evolutionary fate of the gene sequence after the capture event, we analyzed the location of TEs and genes on the chromosome with the capture events. The 2009 capture events where both genes and TEs were located on chromosomes were shown in the Circos (Fig. 2C). Among these events, the 619 pairs of TEs and genes were located in different subgenomes, and 1390 pairs were in the same subgenome. Simultaneously, we analyzed the distribution of donor genes and gene-capturing TEs on chromosomes, and found that the number of both of them was significantly positively correlated with the length of chromosomes, and the longer the chromosomes were, the more gene-capturing TEs and donor genes were distributed (Fig. 2D and Fig. S2). Donor genes and gene-capturing TEs were uniformly and randomly distributed on chromosomes, and the fragments from different genes may be randomly scattered in different locations. According to their relative positions, the gene-capturing TEs and donor genes were divided into four groups: near to each other (within 2 kb), in the same chromosome (>2 kb), in different chromosomes of the same subgenome, and in different subgenome (Fig. 2E). Only one TE was near to gene, and all the remaining TEs carrying sequence moved from the surrounding of the captured genes. Most of them chose to go to different chromosomes but in the same subgenome, and only 5.36% of TEs remained on the same chromosome at positions far from the genes. The capture events made the sequence widely, evenly, and disorderly distribute in the genome, so that different locations in chromosomes had the opportunity to store important information. We found a gene, *BnaC07g44350D*, which was captured 602 times, and it was the most frequently captured gene in the *B. napus* genome. Just on the C_n_07 chromosome where the gene was located, it was captured by 35 TEs, and all TEs carried sequences away from the gene and its flanks. We further analyzed the gene and found that it had three conserved motifs alternating on the DNA sequence, which may be easily recognized and captured by TEs (Fig. S3). We identified the complete and conserved domain of this gene through the Pfam (PF03321) and CDD websites, and the result indicated it was a member of the *GH3* gene family. As primary auxin-responsive genes, GH3 genes were closely related to plant growth and development, and resistance to drought stress [28]. In addition, we identified a 194-Mb pseudogene sequence in the whole genome of *B. napus*, and found that more than half (59%) of the gene-capturing TEs were located on them, becoming part of their composition (Fig. S4).

### The siRNA abundance of donor genes and gene-capturing TEs changed rapidly during allopolyploidy

**Figure 3:**
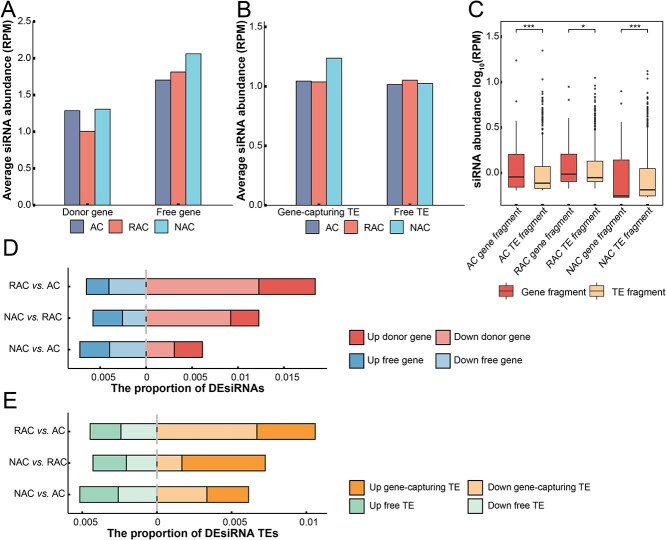
The siRNA abundance of gene-capture events in three genotypes of *B. napus*. (A) Average siRNA abundance of donor and free genes. (B) Average siRNA abundance of gene-capturing and free TEs. (C) The siRNA abundance of captured fragments in gene-capturing TEs and donor genes. (D) The proportion of donor and free genes was regulated by DEsiRNA. (E) The proportion of DEsiRNA in gene-capturing and free TEs during the process of allopolyploidy.

The 24 nt-siRNA was abundant in plant genomes, and they played the key role in the RdDM pathway mediating *de novo* DNA methylation and the regulation of germ cell development [12]. To further explore the effects of epigenetic modification on gene capture of allopolyploid, we performed an in-depth analysis of siRNA in *B. napus* genome. Small RNA-seq was performed on natural *B. napus* (NAC), resynthesized *B. napus* (RAC), and their diploid ancestors *B. oleracea* (C_o_C_o_, maternal) and *B. rapa* (A_r_A_r_, paternal), and the small RNA-seq data of *B. oleracea* and *B. rapa* were mixed in equal ratio to construct the *in silico* ‘hybrid’ (AC). Using *B. napus* Darmor v5 genome [24] as the reference genome, we detected that the siRNA abundance of donor gene was lower than that of the free gene (Fig. 3A), and that of gene-capturing and free TEs was similar in the three genotypes (AC, RAC, NAC) except for the gene-capturing TEs of NAC (Fig. 3B). Moreover, we compared the siRNA abundance transcribed from the same gene fragment between each capture event of donor gene and gene-capturing TE. The siRNA abundance of gene fragments was significantly influenced by the capture events, and it decreased significantly after gene capture in gene-capturing TEs of AC and RAC genotypes, while it increased in NAC genotype (Fig. 3C).

**Figure 4:**
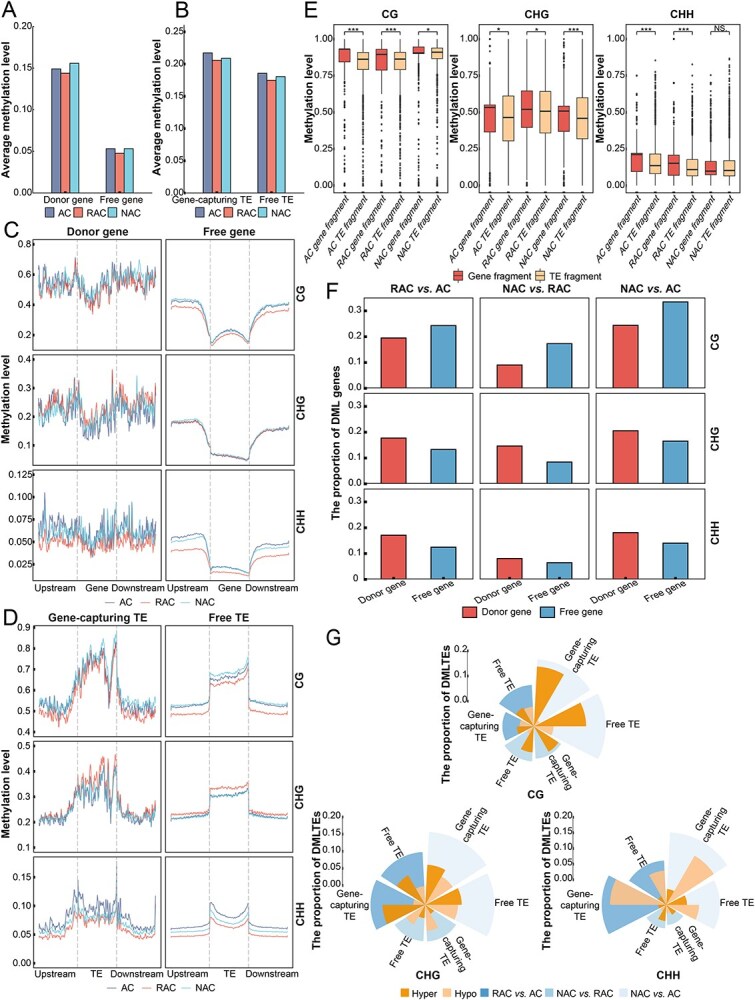
The DNA methylation of gene-capture events in three genotypes of *B. napus*. (A) Whole methylation levels of donor and free genes. (B) Whole methylation levels of gene-capturing and free TEs. (C) DNA methylation profiles of donor genes, free genes, and their upstream and downstream 2 kb. (D) DNA methylation profiles of gene-capturing TEs, free TEs, and their upstream and downstream 2 kb. (E) The CG, CHG, and CHH methylation of captured fragments in gene-capturing TEs and donor genes. (F) The proportion of DML in donor and free genes. (G) The proportion of DML gene-capturing and free TEs.

To explore the effect of epigenetic modification on gene capture in the allopolyploidy process, we further analyzed the differential expressed siRNA (DEsiRNA) among three genotypes of *B. napus*. We found 6 DEsiRNAs in RAC versus AC, 4 in NAC versus RAC, and only 2 in NAC versus AC. The proportion of DEsiRNA in donor genes was the highest in RAC versus AC and the second in NAC versus RAC, and the proportion of DEsiRNA in the two groups was higher than that in free genes, especially siRNA with decreased expression levels (Fig. 3D). During the early establishment (RAC vs AC) and gradual evolution (NAC vs RAC) of polyploid, the corresponding siRNA on the donor genes would respond rapidly, decrease the abundance, and weaken the repression. From a long evolutionary perspective (NAC vs AC), the proportion of genes affected by DEsiRNA was similar between donor and free genes. We also found a similar pattern in the comparison of gene-capturing and free TEs, and the DEsiRNA of gene-capturing TEs was largest in the early establishment of allopolyploid (Fig. 3E).

**Figure 5:**
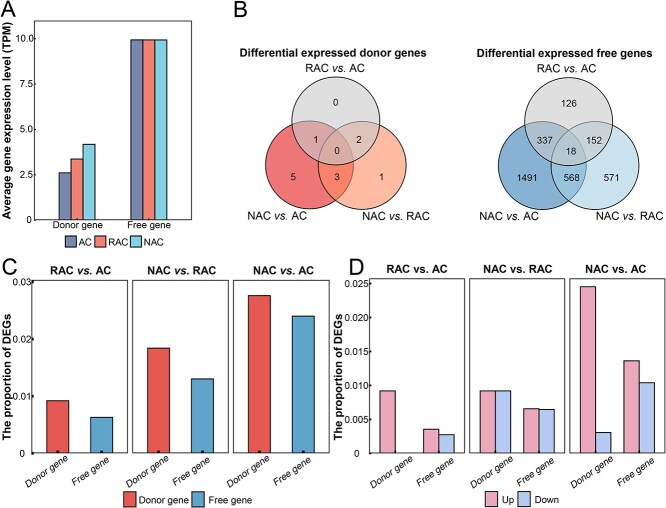
Gene expression levels in capture events during allopolyploidization. (A) Average expression levels of donor and free genes in *B. napus*. (B) The overlap of differential expressed donor and free genes among three comparison groups. (C) The proportion of DEGs to donor and free genes in the three comparison groups. (D) The proportion of expression level up and down of DEGs in donor and free genes.

### The proportion of captured genes and gene-capturing TEs undergoing differential methylation level was higher in the early establishment of allopolyploid than in the stage of gradual evolution

The siRNA may mediate DNA methylation through the RdDM pathway, and the changes of siRNA abundance can affect the methylation level of corresponding DNA sequences. Relying on the methylation database from three genotypes of *B. napus* [27], we further explored the association between epigenetic modification and capture events. The methylation levels of donor genes were nearly three times higher than that of free genes (Fig. 4A), whereas the siRNA abundances of donor genes were lower in *B. napus* capture events (Fig. 3A), and no significant positive correlation was observed between methylation level and siRNA abundance. With the methylation levels of CG, CHG, and CHH (where H = A, T, or C) as the horizontal axis, the proportion of methylation levels distributed in different ranges was calculated (Fig. S5A). The free gene was enriched in low methylation levels in three contexts, while the distribution of CG and CHG methylation in the donor gene showed bimodal. Except at low methylation level (≤10%), enrichment of donor genes was also observed at CG methylation levels >80% and CHG methylation levels between 30% and 50% (Fig. S5A). The donor genes may be significantly silenced by other factors besides their own transcribed siRNA. Consistent with the epigenetic modification of donor gene, the whole methylation of the gene-capturing TEs was higher than free TEs (Fig. 4B), although there was no significant difference in siRNA abundance (Fig. 3B). In the distribution of three context methylation levels, the absolute advantage of the number of CHG and CHH in the low methylation levels (≤10%) of the gene-capturing TEs compared to the free TEs was not found (Fig. S5B). We compared the methylation of donor and free genes and their upstream and downstream, and found that the donor genes and their flanks had higher methylation levels than free genes (Fig. 4C). The donor genes and their flanks had similar methylation levels, especially CHH methylation, while the free genes had lower methylation levels than their flanks. The methylation level of gene-capturing TEs was higher than that of free TEs, with similar methylation levels of two types of TE flanks (Fig. 4D). The levels of DNA methylation on gene fragments were different before and after capture events. The methylation modification levels of captured fragments in the TEs were significantly lower than that in the genes prior to the occurrence of capture events in the AC and RAC genotypes, while this change in NAC was preferred to be inconsistent in CG, CHG, and CHH context (Fig. 4E).

From the time of allopolyploid formation, whole DNA methylation levels of the donor genes and gene-capturing TEs decreased first and then increased, and the RAC had the lowest epigenetic modification level (Fig. 4A and B). In the early establishment period of allopolyploid, the control of capture events by epigenetic modification may be temporarily relaxed. We identified donor and free genes with differential methylation level (DML, Fig. S6). Donor and free genes showed large methylation changes in the early stage of allopolyploid establishment (RAC vs AC), and the proportion of differences decreased during gradual evolution (NAC vs RAC, Fig. 4F). In CG methylation of three comparison groups, the proportion of free genes undergoing differential methylation was higher, while the donor genes were more differential methylation in CHG and CHH methylation (Fig. 4F). The proportion of DML genes increased with the time of polyploid genome formation in CG methylation of gene-capturing TEs, whereas the CHG and CHH decreased first and then increased (Fig. 4G). In CHG and CHH, the methylation of gene-capturing TEs at the early establishment was drastically changed, and was slightly corrected during the subsequent long evolution, while the proportion of DML gene-capturing TEs between NAC and AC was similar to that at the time of early establishment (RAC vs AC).

**Figure 6:**
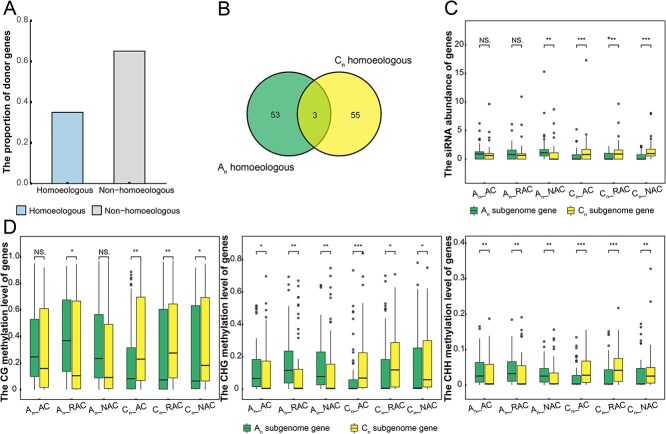
Epigenetic modification profiles of homoeologous genes with gene capture. (A) The proportion of donor genes in homoeologous and nonhomoeologous genes. (B) The overlap of homoeologous gene pairs between A_n_ and C_n_ subgenomes where the donor gene was located. (C) The siRNA abundance of homoeologous genes. (D) The CG, CHG, and CHH methylation levels of homoeologous genes. In (C) and (D), the horizontal coordinate represented the A_n_/C_n_ subgenome where the donor gene was located of homoeologous gene pair in the different genotypes (Wilcoxon test; ^*^P < 0.05, ^**^P < 0.01, ^***^P < 0.001).

### More and more donor genes differentially expressed during allopolyploidization

Plant genomes can change epigenetic modifications to regulate gene expression levels. During allopolyploidization, the newborn genome shock changed epigenetic modifications, and gene expression levels were affected. We further explored the level of gene expression in gene capture events of *B. napus*. With the increase of genome formation time, the expression levels of donor genes were upregulated in the three genotypes, but they were significantly lower than that of free genes (Fig. 5A). We analyzed the differential expressed donor and free genes during allopolyploidization in *B. napus*, and 12 donor genes and 3263 free genes were identified as differential expressed genes (DEGs, Fig. 5B). The proportion of differential expressed donor and free genes increased with the time of polyploid formation, and the proportion of DEGs in donor genes was higher than that of free genes (Fig. 5C). The expression of DEGs was more upregulated than downregulated at all comparison groups, especially the upregulated donor genes had absolute advantages in RAC versus AC and NAC versus AC (Fig. 5D). The donor genes were more affected than the free genes in the allopolyploidy process, although they were small in number and low in expression.

### The siRNA crosstalk enhanced the methylation levels of donor genes

In order to further reveal the effects of gene capture on epigenetic modification in allopolyploid, we performed deeper research on donor genes in homoeologous gene pairs. There were 101 040 genes in total in *B. napus* genome, with 29 976 homoeologous gene pairs (59 952 genes) and 41 088 nonhomoeologous genes. Among 326 donor genes, 35% (114 genes) were homoeologous genes and 65% (212 genes) were nonhomoeologous genes (Fig. 6A). Compared with homoeologous genes that were common in ancestral species, nonhomoeologous genes seemed to be more easily captured. This may be due to the fact that nonhomoeologous genes had fewer functions necessary for life activities than homoeologous genes and less influence on the phenotype, and purifying selection of them were more relaxed.

Homoeologous genes had highly consistent sequences, often originated from a common ancestor, and performed similar functions in the genome. To investigate the gene epigenetic modification changes that occurred due to the TE intervention, we drew the Venn map of homoeologous gene pairs based in the subgenome of donor genes (Fig. 6B). The donor genes of 56 homoeologous gene pairs were located in the A_n_ subgenome, 58 in the C_n_ subgenome, of which donor genes of three homoeologous gene pairs (6 genes) were in the A_n_ and C_n_ subgenomes. To further analyze the siRNA abundance and DNA methylation levels, homoeologous gene pairs with different capture states in the A_n_ or C_n_ subgenome only (A_n_:53, C_n_:55) were divided into six classes based on the ‘genotype_subgenome’ in which the donor genes were located (Fig. 6C and D). Each class contained two groups showing the epigenetic modification level of genes in A_n_ and C_n_ subgenomes of homoeologous gene pairs in this class. Interestingly, either the median levels of siRNA abundance or CG, CHG, and CHH methylation indicated that epigenetic modification of A_n_ subgenome genes was higher than that of C_n_ subgenome when homoeologous gene pairs of donor genes were in A_n_ subgenome. Moreover, when the donor genes were located in the C_n_ subgenome, the siRNA abundance and DNA methylation levels were significantly higher than that of their homoeologous genes in A_n_ subgenome. The above results indicated that epigenetic modification levels of donor genes were higher than those of their uncaptured homoeologous genes, and gene capture was related to asymmetric epigenetic modification of homoeologous gene pairs in the subgenome. Furthermore, whether the homoeologous donor genes were located in the A_n_ or C_n_ subgenome, the median methylation level of the donor gene groups always was the highest in the RAC genotype at the early stage of allopolyploid establishment, and that of the homoeologous gene groups that was not captured tended to stabilize. The DNA methylation of the homoeologous donor genes fluctuated, and it was more intensely regulated by epigenetic modification during allopolyploidy. However, it should be noted that we did not find differences in gene expression levels of all groups (Fig. S7). This may be because of the fact that the donor gene expression levels were low (Fig. 5A), and the higher proportion of homoeologous donor genes silenced, which caused insignificant changes in expression levels in the process of allopolyploidy.

## Discussion

TEs regulate the neighboring gene expression and change plant phenotypes through insertion and epigenetic modification. This has been a research hotspot, and deeply studied in rice and Arabidopsis [29, 30]. In contrast, the effects of epigenetic modification of TEs on gene capture and donor gene expression remain to be explored. Especially in the process of allopolyploidy, TEs are activated and expanded due to genomic shock, but the research on TE capturing genes in this period is still a blank. Here, we used allotetraploid *B. napus* to analyze the characteristics of capture events and the effects of epigenetic modifications on capture, and to explore in depth the new mechanisms of gene-capture events regulated by epigenetic modifications during allopolyploidization.

### Genome stored a large number of gene fragments captured by TEs, providing opportunities for low-cost mutations in *B. napus*

The evolution of species is closely related to genome changes, and genome variation leads to the formation of new traits [31]. TEs can jump and insert into the genome, change chromosome structure, and they are often considered to be an important driving factor for genome evolution [10]. Gene capture by TEs allowed genetic information to spread across the genome in the form of fragments that may lead to new traits. Some studies had used TEs that carry gene fragments to integrate into genome and change gene expression, realizing the construction of therapeutic cell systems [32]. Hybridization of two genomes and polyploidization resulted in the formation of a new allopolyploid genome, with TEs activated and expanded, and the genome encountered drastic shock in the natural world [3]. In *B. napus*, capture events were few in number but real, and occurred uniformly across the genome. We found that a large number of captured exons were located in UTRs (Fig. 1C), and the expression levels of donor genes were much lower than that of free genes (Fig. 5A). Homoeologous genes derived from different ancestral species may contain more functional and phenotypic related genes than nonhomoeologous genes. Compared with free genes, more donor genes were nonhomoeologous genes, and the natural selections were more relaxed (Figs 2B and 6A). The donor genes may not be preferred for strong function, and protein-encoding sequences with high expression were the secondary purpose of TEs to capture gene exons. TEs carried the captured sequence away from the donor genes and even traveled to another subgenome (Fig. 2C and E). Meantime, the sequence of donor genes multiplied in the genome, retaining and storing genetic information (Fig. 2D). When the 3′ termination signal of TEs *Helitron* was destroyed, it can achieve capture by peel-and-paste while keeping the gene sequence unchanged [21]. Tens of thousands of gene fragments and some even almost complete copies had been captured in maize [33, 34]. This made the genome pay a small evolutionary cost (the original gene sequence and structure were not destroyed) but created a lot of number of new loci of gene copy sequences to mutation.

### TEs hid in the genome after the gene-capture event, and favored the formation of new genes and transcripts

Allopolyploidy led to activation and genome shock in plants, and the new genomes were dramatically challenged. The jumping and expansion of TEs affected genome stability and destroyed the gene expression, which made a deep influence and evolutionary pressure in genome. To reduce the effects of TE expansion, the genome initiated multiple epigenetic modifications to silence and inhibit TEs [35]. At the same time, TEs were actively searching for counterdefense and antisilencing mechanisms to preserve themselves by inserting into safe locations, providing value to the host, and capturing genes [36]. Gene capture made the TEs and genes carry the same fragments, and the gene-capturing TEs were different to be identified and distinguished with complete accuracy. Epigenetic modifications originally used to silence TEs may be affected by siRNA crosstalk targeting genes with the same fragments [22]. When genes had important functions, it was difficult for host to silence TEs without affecting its own gene expression, and TEs were thus hidden in the genome to escape harsh repression in some extent.

TEs carrying gene fragments may also have new missions in the genome. More than half of the gene-capturing TEs in *B. napus* were involved in the pseudogene composition (Fig. S4). Gene-capturing TEs pointed to the origin of pseudogenes, and captured fragments evolved with pseudogenes and played important functions in affecting chromatin and genome structure [37]. Pseudogenes derived from TEs can integrate resources to obtain multiple expression patterns and functions, and use noncoding RNA mechanisms to achieve regulation of parental gene expression levels, which provide opportunities for evolution as substrates for selection [38]. The pseudogenic *HBBP1* played an essential role in human-specific erythropoiesis through the regulation of the key regulator *TAL1* [39]. In addition, 35.25% of the gene-capturing TEs in *B. napus* carried more than one exon fragment, and gene-capture event disrupted the order and location of exons so that the chimeras of different genes were transcribed in the TEs. In maize, capture events of *Helitron* TEs had been found to generate gene chimeric transcripts and alternatively spliced transcripts, which may contribute to the formation of new phenotypes [40]. What’s more, highly similar genes are easily deleted when they are in structural and functional entanglement with each other [41]. Gene-capturing TEs may act as a mediator for this entanglement, and dosage-sensitive genes were negatively affected by a large number of segmental duplications and they were eliminated [42]. The relationship between TE and genome was not a simple confrontation, and they gradually formed a complex relationship of promotion, competition, cooperation, and parasitism in countless days and nights of mutual struggle.

### Regulation of donor gene expression levels may benefit from relaxed gene-capturing TE repression and crosstalk in the early stage of allopolyploid establishment

To maintain genome stability, plants often silence TEs by various methods such as DNA methylation. The RdDM pathway is an important source of *de novo* DNA methylation in plants, in which siRNAs are loaded into ARGONAUTE (AGO4) and recruited to direct DOMAINS REARRANGED METHYLASE2 (DRM2) to facilitate methylation [43]. In rice, siRNA abundance and DNA methylation levels were positively correlated, and the elimination of siRNA accumulation can substantially decrease methylation levels [44]. We found that donor genes had lower siRNA abundance but higher methylation levels than free genes in *B. napus* gene-capture events (Fig. 3A and 4A). This may be the result of siRNA crosstalk, where the siRNAs in the RdDM pathway were shared between the donor gene and the gene-capturing TEs [22]. The siRNA that was supposed to target the TEs bound to the homoeologous donor gene fragment and mediated gene methylation. Epigenetic modifications can not only silence TEs and maintain genome stability, but also affect gene expression and contribute to the formation of new phenotypes [45]. The expression levels of donor genes in three genotypes of *B. napus* were significantly lower than those of free genes (Fig. 5A), and the genome preferentially selected for control active TEs, maintained genome stability, and consequently lost the expression level of some genes.

The epigenetic modification levels of donor genes and gene-capturing TEs in RAC were lower than that of AC and NAC genotypes (Figs 3A and B and 4A and B). Allopolyploidization leads to changes in epigenetic modification of the genome and activation of TEs. During the early establishment of allopolyploid (RAC vs AC), the siRNA abundance of TEs was more differential expression downregulated (Fig. 3E), and the expression levels of donor genes were differentially upregulated (Fig. 5D), by which the genome briefly weakened the control of TEs, thereby reducing the confusion of crosstalk on gene expression and recovering the expression levels of some genes. The *B. napus* genome may achieve regulation of donor gene expression levels in allopolyploidization, relying on the changes of epigenetic modification of genes captured by TEs.

## Materials and methods

### Plant materials

*Brassica napus* (natural and resynthesized allotetraploid) and its diploid parents (*B. rapa* and *B. oleracea*) were used as plant materials. The natural *B. napus* was ‘Darmor’ (A_n_A_n_C_n_C_n_, NAC), while the resynthesized *B. napus* ‘HC-2’ (A_n_A_n_C_n_C_n_, RAC) was formed by hybridization and polyploidization of *B. oleracea* ‘3YS013’ (C_o_C_o_, maternal) and *B. rapa* ‘9JC002’ (A_r_A_r_, paternal). The seeds were planted in a light incubator with 23°C and 16-h/8-h day/night and regularly watered with Hoagland’s nutrient solution. At 1 month of age, the leaves of healthy plants were harvested and frozen in liquid nitrogen and then stored in −80°C for sequencing.

### Identification of gene capture

Using Extensive *de novo* TE Annotator (EDTA v1.8.2) [46] to annotate TEs in the *B. napus* Darmor v5 genome [24] and relying on the HelitronScanner software in the pipeline, we obtained high-quality *Helitron* dataset [26]. Genes and TEs with physical intersections in the genome were filtered out, and the remaining gene exon sequences were compared with TE sequences using BLASTN. With strict standard *E-value* < 1 × 10^−40^, the similar TEs and genes were screened out one by one. The gene corresponding to a TE with the highest BLASTN bit score was identified as the donor gene of the TE. When more than one gene exon was identified with the same *E-value* and bit score for similarity to the TEs, they were all retained. According to the number of capture events on the TEs, they were classified into single and multiple gene-capturing TEs.

### Sequence characteristics of capture events

The length and number of donor and free gene exons were counted using the Wilcoxon test (***P < 0.001). The proportion of captured exons that were UTRs and coding sequences (CDS) was calculated in all capture events. Python scripts were used to calculate the density of genes, gene-capturing TEs, and free TEs per Mb on the chromosome, and the Circos diagram was drawn by TBtools [47]. In order to explore the sequence characterization of captured exons and gene-capturing TEs, we used the MEME website (https://meme-suite.org/meme/index.html) for sequence motif enrichment and showed the top five results according to *E-value*.

To understand the evolutionary pressure on genes from capture events, we classified previously identified homoeologous gene pairs in *B. napus* into donor gene pairs and free gene pairs [27]. Each donor gene pair contained at least one donor gene, while it was considered a free gene pair only when both homoeologous genes were free genes. The Ka, Ks, and Ka/Ks values of donor and free homoeologous gene pairs were calculated using TBtools [47]. We counted the length of chromosomes, and the number of donor genes and gene-capturing TEs located on chromosomes. Based on the relative positions, the subgenome relationship between genes and TEs in the capture event was shown by a Circos diagram. The position relationship between the donor gene and gene-capturing TE was further analyzed, and the proportion of gene-capturing TEs with genes: near to each other (within 2 kb), in the same chromosome (> 2 kb), in different chromosomes of the same subgenome, and in different subgenome four groups were calculated, respectively. We also used PseudogenePipeline [48] to annotate the pseudogenes of *B. napus* genome, and identified the gene-capturing TEs overlapping with the pseudogene sequences by BEDTools v2.29.2 [49].

### Analysis of siRNA abundance, DNA methylation, and gene expression levels

To explore epigenetic modification levels and gene expression levels of gene-capture events at different evolutionary stages, we retrieved small RNA-seq, whole-genome bisulfite sequencing (WGBS) and RNA-seq data of *B. napus* and its diploid parents from previous studies [26, 27]. Based on a 1:1 ratio, the filtered clean data from *B. rapa* (A_r_A_r_) and *B. oleracea* (C_o_C_o_) were mixed and obtained *in silico* ‘hybrid’ (AC) multiomics data for subsequent analysis. The siRNA, DNA methylation, and gene expression levels were mapped to the *B. napus* genome Darmor v5 [24], and contained three biological replicates. After filtering out tRNA, miRNA, rRNA, snRNA, and snoRNA, 24 nt-siRNA reads were retained, and the expression levels were normalized to reads per million (RPM). BatMeth2 [50] was used to calculate methylation levels in genes, TEs, and their upstream and downstream 2 kb in capture events. Methylation levels were divided into 10 groups, and the proportion of the number of genes and TEs belonging to the different methylation groups was drawn. The captured gene fragments were accurately located, and the siRNA abundance and DNA methylation levels of the captured fragments in the gene-capturing TEs and donor genes were calculated and compared for each pair. The overlap of homoeologous gene pairs was drawn in accordance with the subgenome of donor gene location, and homoeologous gene pairs with only one donor gene were further identified for siRNA abundance and DNA methylation levels. Simultaneously, the gene expression levels were normalized in terms of transcripts per million reads (TPM).

### Differential epigenetic modification and gene expression

The DEGs among three genotypes of *B. napus* were identified by DESeq2 (|Log_2_(fold change)| ≥ 1, *P* ≤ 0.001. The DEsiRNA and DML genes and TEs were interrogated by Student’s *t*-test (*P* < 0.05), and their proportion in the classification was calculated, respectively.

### Statistical analysis

In this study, graph drawing (histogram, box plot, scatter plot, and density plot) and statistical tests (Student’s *t*-test, Wilcoxon test) were realized by R.

## Supplementary Material

Web_Material_uhaf028

## Data Availability

The raw data that support this study are available in the National Center for Biotechnology Information (NCBI) Sequence Read Archive (SRA) database (RNA-seq, SRR13302173-13302184; Small RNA-seq, SRR24296930-24296941; WGBS, SRR13306925-13306936).
