# Supplementary material for: Epigenetic modification brings new opportunities for gene capture by transposable elements in allopolyploid Brassica napus
Source: Hortic Res. 2025 Jan 27;12(5):uhaf028. doi: 10.1093/hr/uhaf028 (PMC11986588; doi:10.1093/hr/uhaf028)
Supplement: Web_Material_uhaf028 [file web_material_uhaf028.zip › Table S1. The sequence composition of gene-capturing and free TEs.docx]

**Table S1. The sequence composition of gene-capturing and free TEs.**

|  | Gene-capturing TE | Free TE |
| --- | --- | --- |
| TE number | 1793 | 348848 |
| Total sequence length (bp) | 2229159 | 143321509 |
| Minimum length (bp) | 92 | 80 |
| Maximum length (bp) | 19864 | 120159 |
| Average length (bp) | 1243 | 411 |
| GC content (%) | 31 | 30 |
