# Supplementary figures and images for: Epigenetic modification brings new opportunities for gene capture by transposable elements in allopolyploid Brassica napus
Source: Hortic Res. 2025 Jan 27;12(5):uhaf028. doi: 10.1093/hr/uhaf028 (PMC11986588; doi:10.1093/hr/uhaf028)

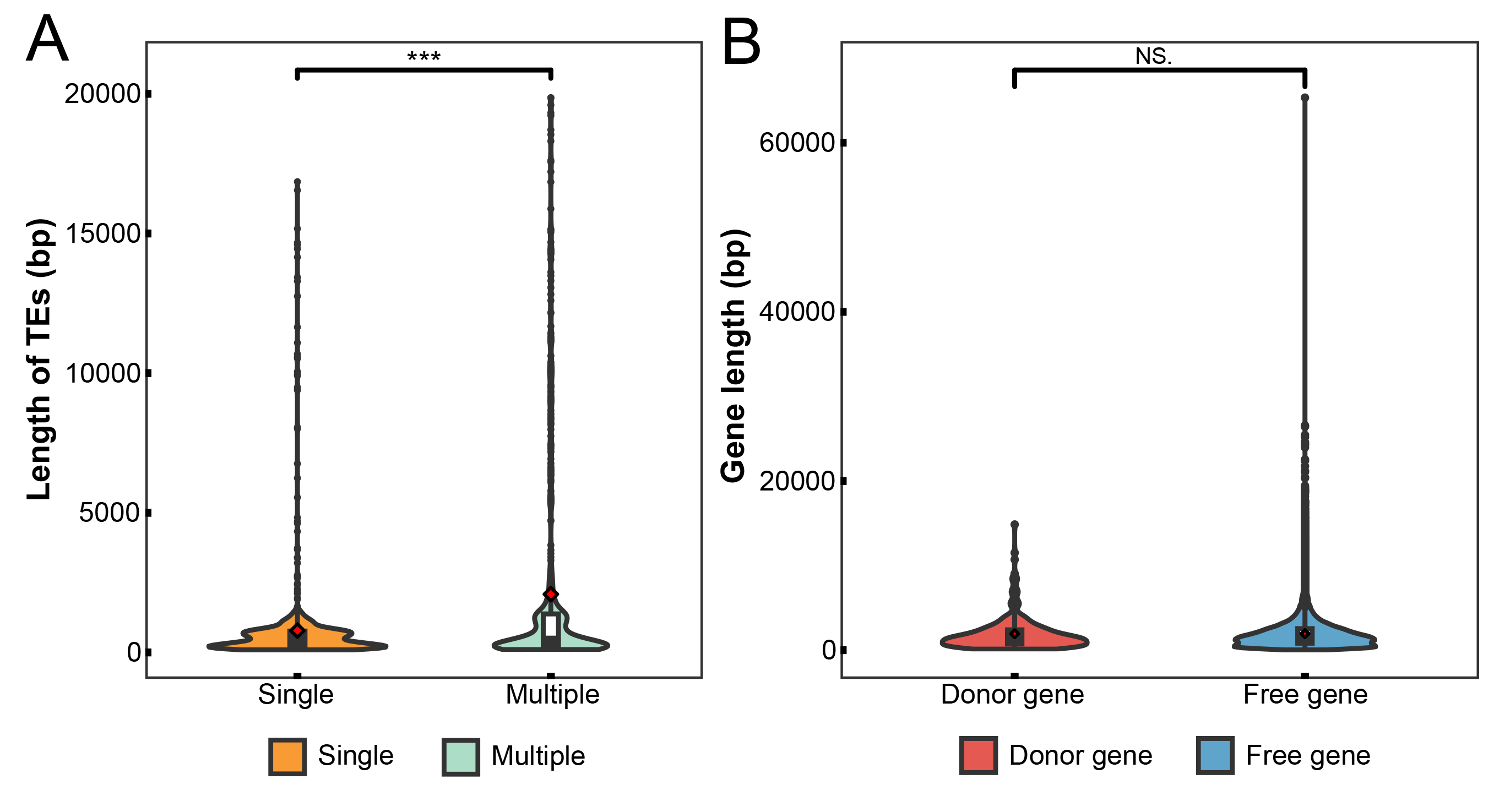

Supplement: Web_Material_uhaf028 [file web_material_uhaf028.zip › Fig. S1.tif]

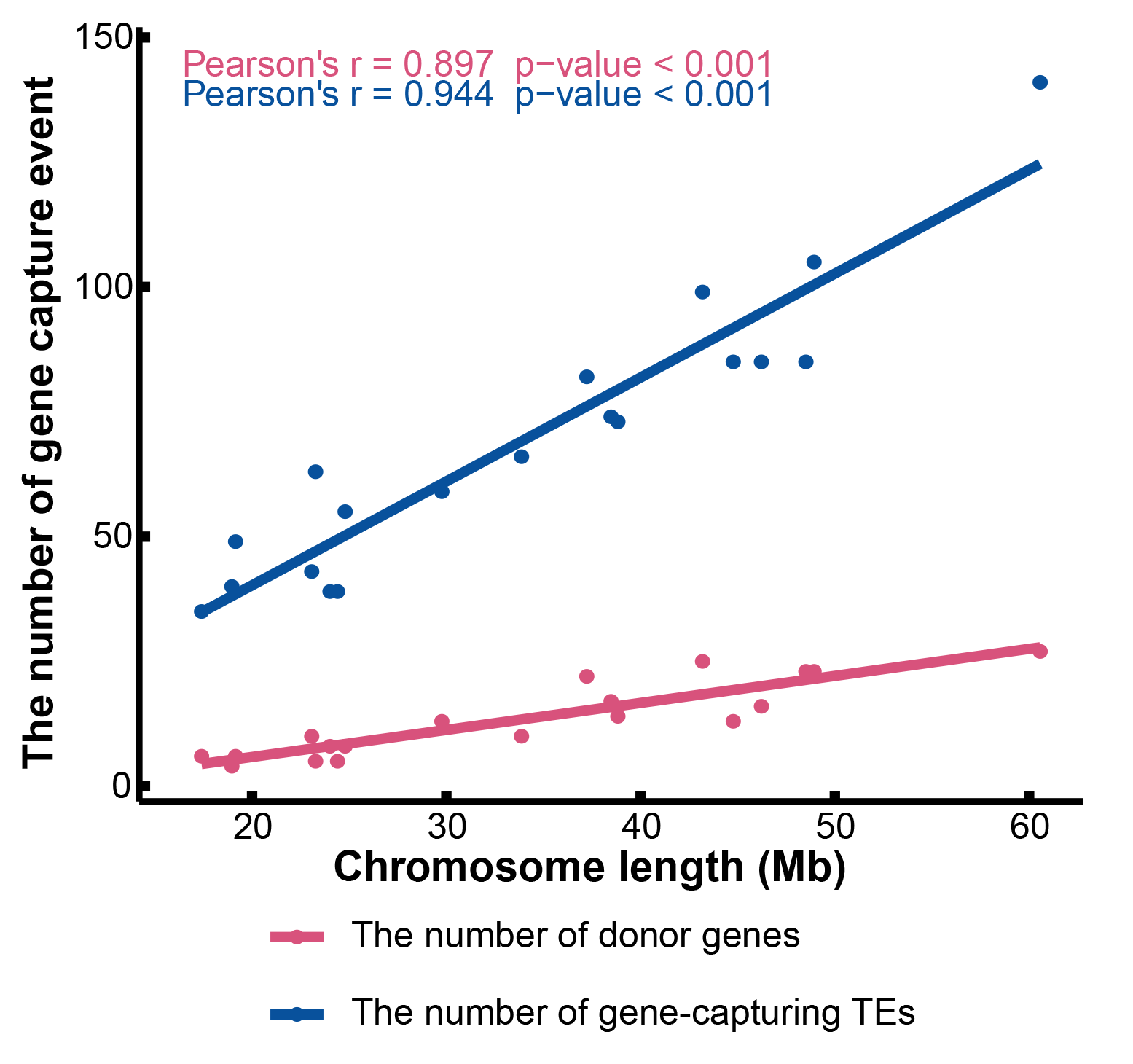

Supplement: Web_Material_uhaf028 [file web_material_uhaf028.zip › Fig. S2.tif]

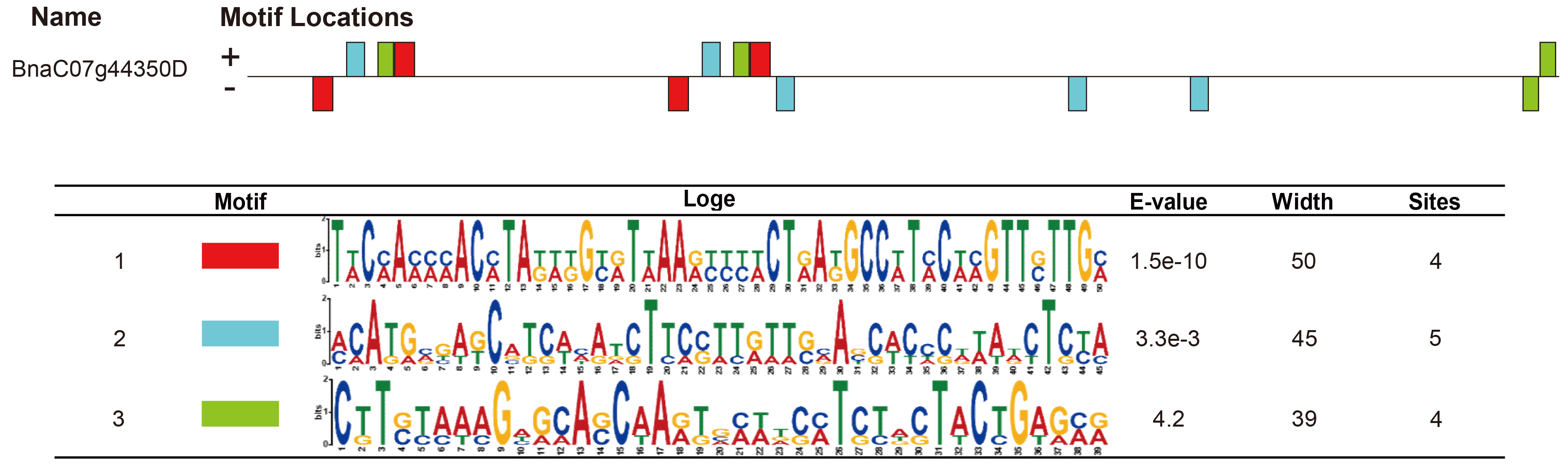

Supplement: Web_Material_uhaf028 [file web_material_uhaf028.zip › Fig. S3.tif]

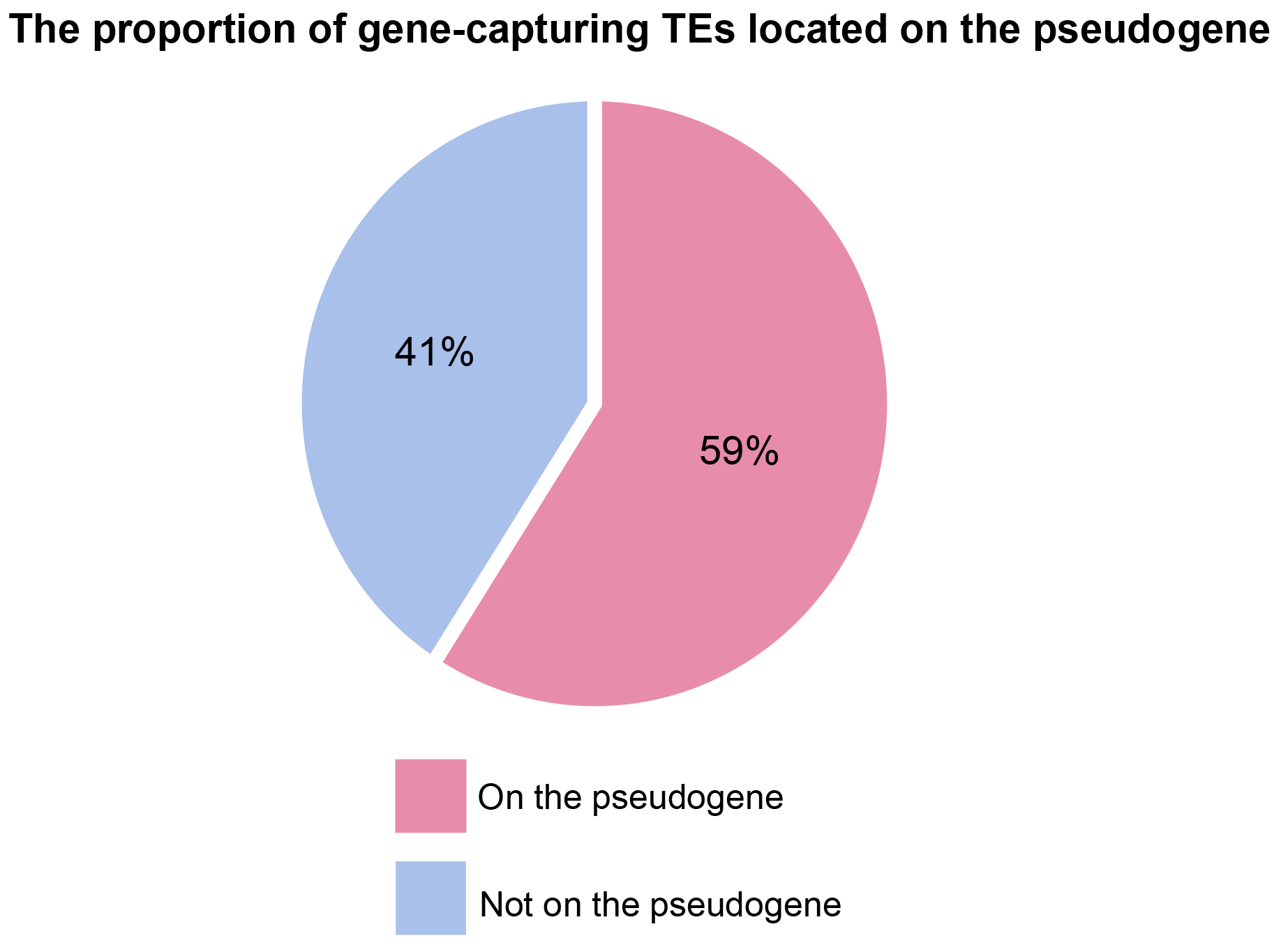

Supplement: Web_Material_uhaf028 [file web_material_uhaf028.zip › Fig. S4.tif]

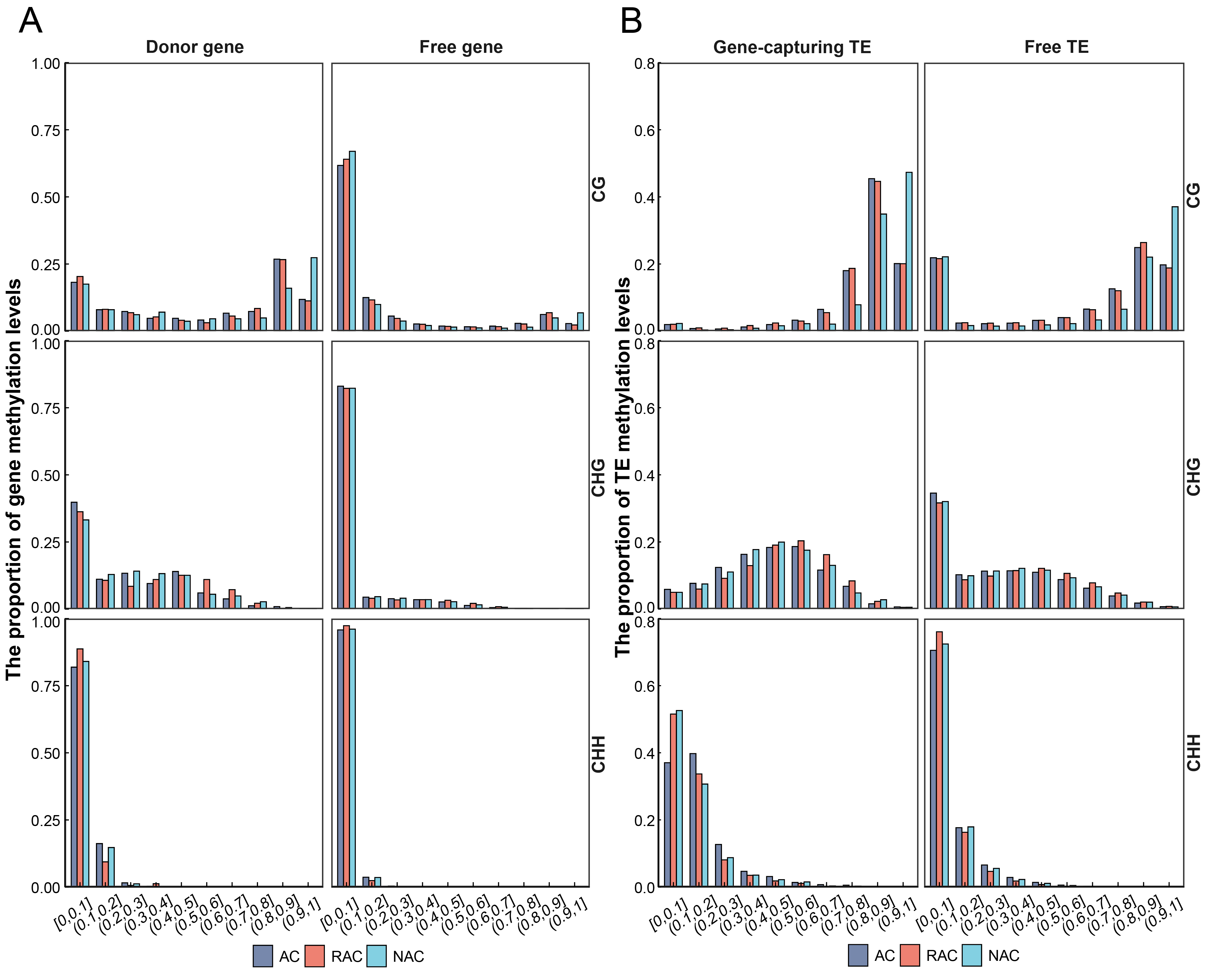

Supplement: Web_Material_uhaf028 [file web_material_uhaf028.zip › Fig. S5.tif]

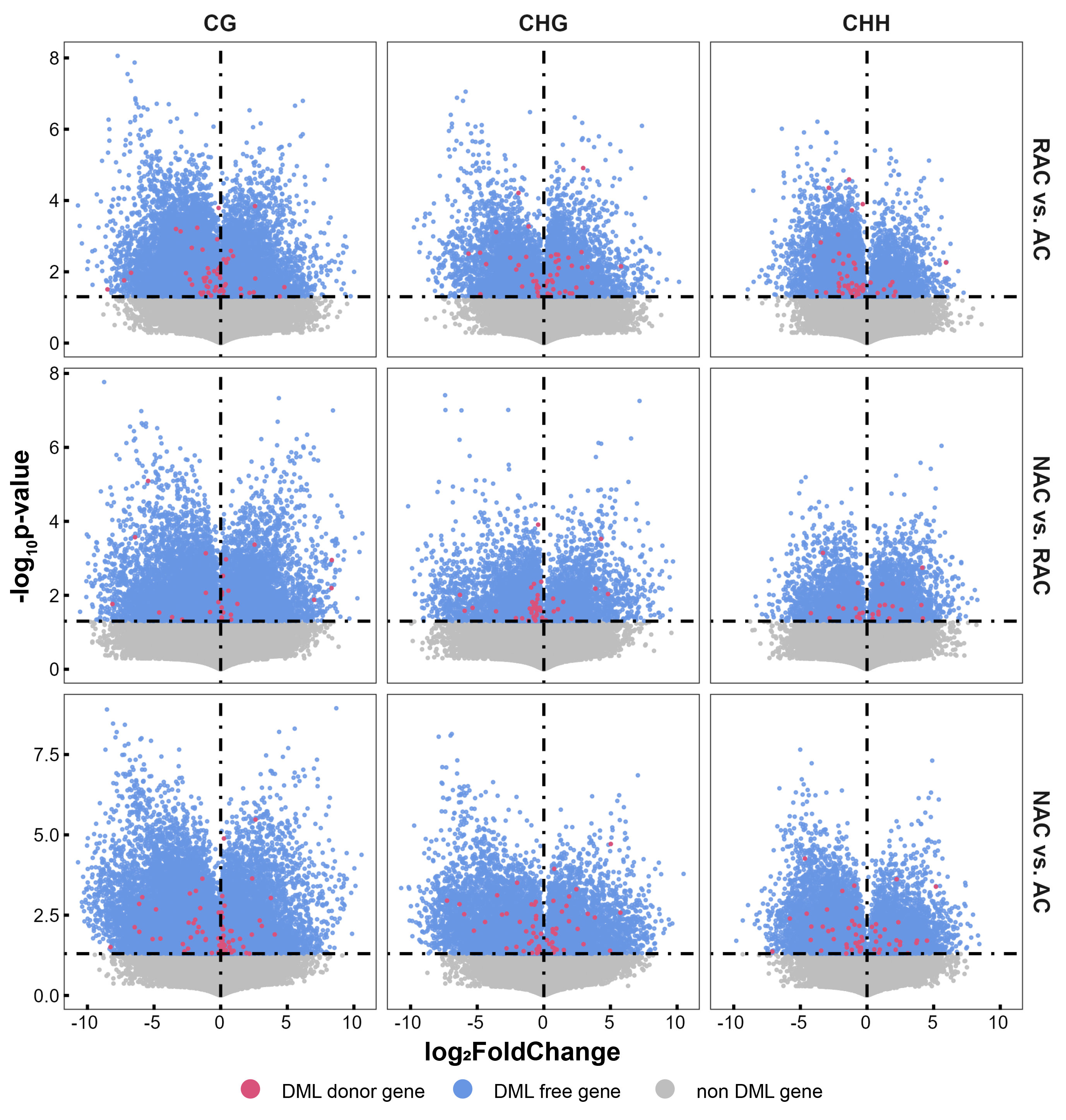

Supplement: Web_Material_uhaf028 [file web_material_uhaf028.zip › Fig. S6.tif]

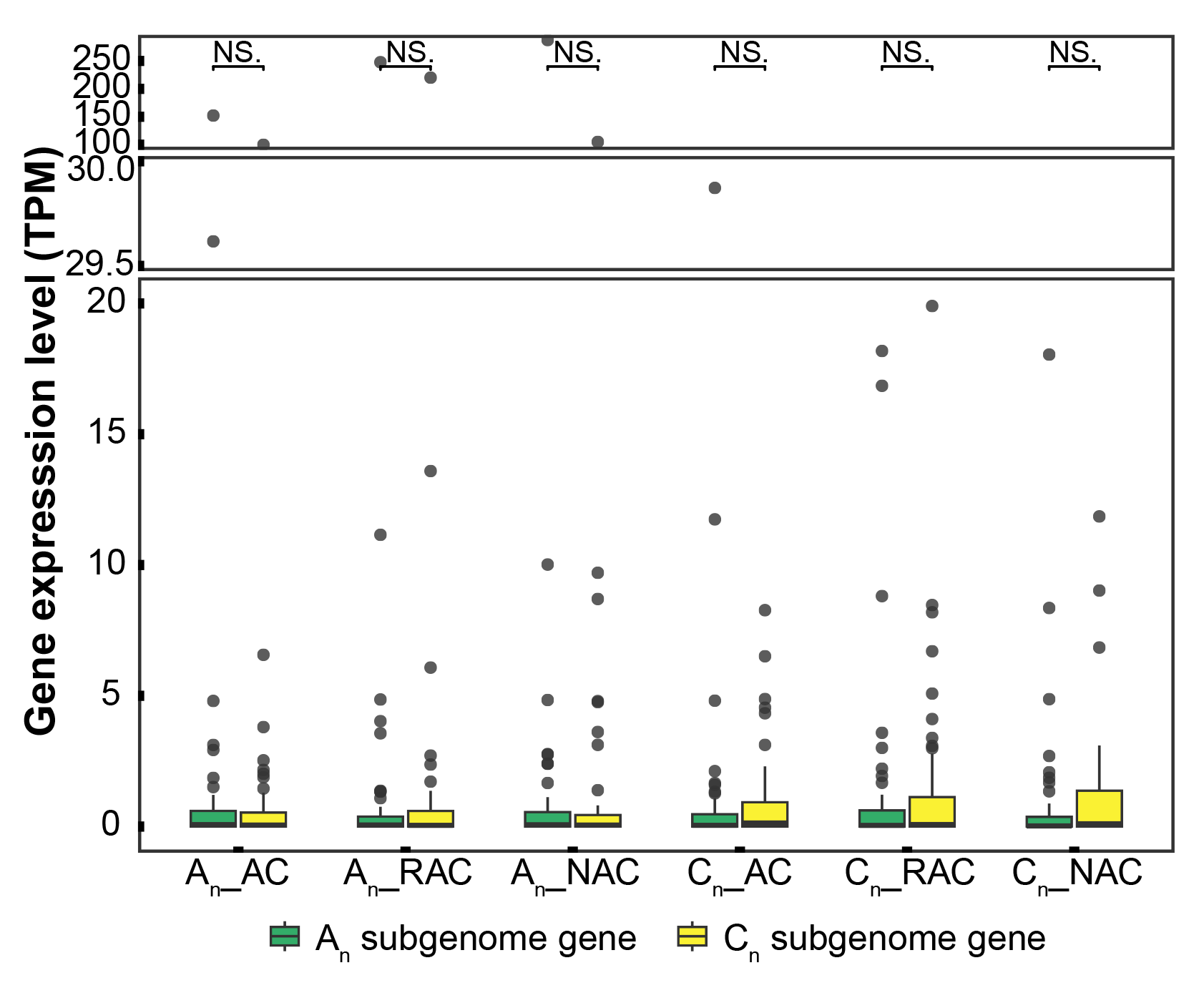

Supplement: Web_Material_uhaf028 [file web_material_uhaf028.zip › Fig. S7.tif]
